# Space–time mapping of wasting among children under the age of five years in Somalia from 2007 to 2010

**DOI:** 10.1016/j.sste.2015.12.002

**Published:** 2016-02

**Authors:** Damaris K Kinyoki, James A Berkley, Grainne M Moloney, Elijah O Odundo, Ngianga-Bakwin Kandala, Abdisalan M Noor

**Affiliations:** a Spatial Health Metrics Group, INFORM Project, Kenya Medical Research Institute/Wellcome Trust Research Programme, Nairobi P.O. Box 43640-00100, Kenya; b Kenya Medical Research Institute/Wellcome Trust Research Programme, Centre for Geographic Medicine Research (coast), Kilifi, Kenya; c Centre for Tropical Medicine and Global Health, Nuffield Department of Clinical Medicine, University of Oxford, CCVTM, Oxford OX3 7LJ, UK; d Nutrition Section, United Nations Children's Fund (UNICEF), Kenya Country Office, UN Complex Gigiri, Nairobi, Kenya; e Food Security and Nutrition Analysis Unit (FSNAU) – Somalia, United Nations Food and Agricultural Organization, Ngecha Road Campus, Nairobi, Kenya; f Warwick Medical School, Health Sciences Research Institute, University of Warwick, Warwick Evidence, Gibbet Hill, CV4 7AL Coventry, UK; g Department of Mathematics and Information sciences Faculty of Engineering and Environment, Northumbria University, Newcastle upon Tyne, NE1 8ST, UK; h Health Economics and Evidence Synthesis Research Unit, Department of Population Health, Luxembourg Institute of Health L-1445 Strassen, Luxembourg

**Keywords:** Malnutrition, Wasting, Bayesian, Space–time model, Seasonality

## Abstract

**Objective:**

To determine the sub-national seasonal prevalence and trends in wasting from 2007 to 2010 among children aged 6–59 months in Somalia using remote sensing and household survey data from nutritional surveys.

**Methods:**

Bayesian hierarchical space–time model was implemented using a stochastic partial differential equation (SPDE) approach in integrated nested Laplace approximations (INLA) to produce risk maps of wasting at 1 × 1 km^2^ spatial resolution and predict to seasons in each year of study from 2007 to 2010.

**Results:**

The prevalence of wasting was generally at critical levels throughout the country, with most of the areas remaining in the upper classes of critical and very critical levels. There was minimal variation in wasting from year-to-year, but a well-defined seasonal variation was observed. The mean difference of the prevalence of wasting between the dry and wet season ranges from 0% to 5%. The risks of wasting in the South Central zone were highest in the Gedo (37%) and Bay (32%) regions. In North East zone the risk was highest in Nugaal (25%) and in the North West zone the risk was high in Awdal and Woqooyi Galbeed regions with 23%.

**Conclusion:**

There was a clear seasonal variation in wasting with minimal year-to-year variability from 2007 to 2010 in Somalia. The prevalence was high during the long dry season, which affects the prevalence in the preceding long rainy season. Understanding the seasonal fluctuations of wasting in different locations and at different times is important to inform timely interventions.

## Highlights

• Bayesian hierarchical space–time model was used to predict prevalence of wasting.
• The rate of wasting was at critical and very critical levels throughout the country.
• There were minimal annual fluctuations of wasting, but a marked seasonality.
• Rainfall and vegetation are important environmental predictors of wasting in Somalia.

## 1 Introduction

Wasting, defined as low weight-for-height, is a strong indicator of mortality among children under the age of five years (Mason et al., 2010). Wasting is thought to develop as a result of acute food shortage and/or disease (Vesel et al., 2010) and is used to assess individuals for supplementary and therapeutic feeding, and to assess emergency humanitarian needs in regions vulnerable to drought, displacement and related causes of food insecurity (Chotard et al., 2010).

Globally, approximately 52 million (8%) children under-five years of age were wasted in 2010, a 11% decrease from an estimated 58 million in 1990 (Chotard et al., 2010). About 70% of the world's wasted children live in Asia where approximately 1 in 6 children (16%) is moderately or severely wasted. In sub-Saharan Africa, 1 in 10 children under the age of five (9%) were wasted in 2010, a prevalence that has barely changed since 1990 (10%) (UNICEF et al., 2013).

A number of risk factors including seasonal food insecurity and environmental conditions have previously been associated with wasting (Engebretsen et al., 2008, Rice et al., 2000, Black et al., 2008, Bhutta et al., 2008, Kinyoki et al., 2015). Wasting is thought to develop over a short period and is reversible with short term intervention (Bhutta et al., 2008). Wasting typically varies seasonally due to variations in climate, disease and food availability (Khara and Dolan, 2014). This leads to potential variation of wasting between populations in normal conditions as well as under drought (Chotard et al., 2010). In Gambia, the prevalence of wasting fluctuated between 4% and 10% between seasons (Tomkins et al., 1986) while in Ethiopia, seasonality did not have a significant effect on wasting in children (Branca et al., 1993).

Thus, the prevalence of wasting determined through nutrition surveys is often highly variable and needs to be interpreted in relation to both emergency factors, climatic anomalies and seasonality which may also vary sub-nationally, to help with planning timely interventions. This information is useful both for assessing the severity of malnutrition at one time and for predicting early how far malnutrition rates are likely to rise with the onset of drought and resulting food insecurity (Chotard et al., 2010). This paper seeks to understand the dynamics of wasting and the role of seasonality among children aged 6–59 months in Somalia using space–time geo-statistical models from 2007 to 2010.

## 2 Methods

### 2.1 Data

The data used for this study were obtained from surveys undertaken by the Food Security and Nutrition Unit (FSNAU) in Somalia from 2007 to 2010. Within this period, FSNAU, in partnership with United Nations Children's Fund (UNICEF), conducted bi-annual seasonal nutrition assessment surveys using Standardized Methodology for Survey in Relief and Transition (SMART) methods, indicators and tools for data collection (Chotard et al., 2010, WHO et al., 2010, FAO/FSNAU-Somalia, 2011). Detailed description of the survey methods are provided elsewhere (Smith, 2006).

Using a multi-stage cluster sampling method across all districts, over 100,000 children were interviewed in approximately 1,100 unique clusters. We undertook a detailed search to establish a set of spatial coordinates for each cluster, which were verified using Google Earth (Google, Seattle, USA) to visually inspect whether the coordinates matched evidence of human settlement. Those settlements for which no reliable sources of coordinates were obtained were excluded from the analysis.

Five environmental covariates associated with vector-borne diseases (Gething et al., 2011) and food security (Grace et al., 2012) were examined for modeling. These were rainfall, enhanced vegetation index (EVI), mean temperature, distance to water, and urbanization. Rainfall and mean temperature were derived from the monthly average grid surfaces obtained from WorldClim database (WorldClim). The EVI values were derived from the MODerate-resolution Imaging Spectroradiometer (MODIS) sensor imagery (Scharlemann et al., 2008) for period 2000–2010 while the urbanization information was obtained from Global Rural Urban Mapping Project (GRUMP) (Balk et al., 2005). All the environmental covariates were extracted from 1 × 1 km^2^ spatial resolution grids to data points. Rainfall, temperature and EVI were summarized to compute seasonal averages using the four main rainy seasons in Somalia.

The data were aggregated at cluster level with the corresponding geographical covariates, year and season of survey. Each record represented a cluster and consisted of the number of children wasted and total children examined together with a list of geographical covariates and the corresponding year and season of survey.

### 2.2 Statistical methods

We began the analysis with the examination of the significant effects of the five environmental covariates. These covariates were selected based on the Bayes Information Criterion (BIC) through best generalized linear approach (McLeod and Xu, 2010). A uniform prior of the model of fixed size implemented in *BIC_γ_* was used.

$$BIC_{\gamma }=D+k\log\left(n\right)+2\gamma log\left(\begin{array}{c} p\\ k \end{array}\right)$$

This is where *γ* is an adjustable parameter, *p* is the number of possible input covariates not counting the bias or intercept term and *k* is the number of the parameters in the model (McLeod and Xu, 2010). We then implemented Bayesian hierarchical space–time models through Stochastic Partial Differential Equations (SPDE) approach using R-INLA library to produce continuous maps of the prevalence of wasting at 1 × 1 km^2^ spatial resolution and predicted to each season of the year from 2007 to 2010 (Rue et al., 2009, Blangiardo et al., 2013). We investigated two main models: the first model focused on the year-season prediction in four main seasons: December–March, the ‘Jilaal’, which is a harsh dry season; ‘Gu’ is the main rainy season from April to June; from July to September is the second dry season, the ‘Hagaa’; and the short rainy season known as ‘Deyr’ from October to November. This is where the year and the corresponding season of survey together were used to define the temporal effects. The main rational for the first model was to estimate the predicted prevalence of wasting by season in each year of survey from 2007 to 2010. In the second model, the year was used to define the temporal effect while the seasons were separately used to define the seasonality effects of wasting within a year. The aim of this model was to determine the effect size of the four seasons in Somalia.

In our analysis, the observed prevalence of wasting in a particular cluster and time is assumed to have a distribution that belongs to the exponential family and the parameter of the family (*ϕ*) is linked to a structured additive predictor *η* through a link function *g*( · ) such that the linear predictor *η* is defined. The hierarchical space–time binomial model of the prevalence of wasting was represented as the realization of a spatial–temporal process of wasting at the survey location, survey date, significant covariates at sampled locations, and the measurement error defined by the Gaussian white noise process. This can simply be denoted as,

$$\eta =\beta_{0}+\beta_{i}Cov+\sum_{k=1}^{K}f_{k}\left(c\right)w_{k}+f\left(•\right)$$

This equation defines a hierarchical model where *β*_0_ is the intercept, *β* denotes the linear regression coefficients for the covariates(*Cov*). The function *f_k_* is the sum of smooth functions defining the random effect of the clusters where the regression coefficients vary with *K* values and *w_k_* is a vector of sampled clusters. *f*(•) is a semi-parametric function defining the spatio-temporal random effect in the model. These components form the non-observable latent field defined as $\theta =\{\beta_{0},\beta ,f\}$ where *β* and *f* are the covariates and smooth functions included in the linear predictor with their appropriate priors (*ψ*).The latent field is characterized by a joint normal Gaussian multivariate distribution with mean 0 and precision matrix *Q*(*ψ*), i.e., $\theta ∼N(0,Q^{-1}\left(\psi \right))$. Each observation *y_i_* depends on a linear combination of a subset of the elements of *θ* defined as:

$$y_{i}|\theta ,\psi ∼p\left(y_{i}|\sum_{j}A_{ij}\theta_{j},\psi \right)$$

This is where *A_ij_* is the generic element of the observed matrix *A* defined by the SPDE approach. The SPDE is formulated as a link between Gaussian random fields (GRFs) and the Gaussian Markov Random Fields (GMRFs) (Blangiardo et al., 2013). The spatio-temporal covariance function and the dense covariance matrix of the Gaussian field are replaced by a neighborhood structure and a sparse precision matrix respectively that together define a GMRF (Blangiardo et al., 2013). This finite-dimensional GMRF that substitutes infinite-dimensional GRF can be expressed as,

$$x\left(u\right)=\sum_{i=1}^{n}\psi_{i}\left(u\right)w_{i}$$

This SPDE-formulation is motivated by computational benefits and also introduces a new class of spatial models (Cameletti et al., 2013). In this SPDE approach, we used a non-stationary model achieved by modifying the SPDE to obtain the GRFs with defined dependence structure and expressed as

$$\left(k{\left(u\right)}^{2}-\Delta \right)\left(\tau \left(u\right)x\left(u\right)\right)=w\left(u\right),\;\;u\in \Omega ,$$

The log *k*(*u*) and log *τ*(*u*) are defined as linear combinations of basis functions,

$$\log\left(\tau \left(u\right)\right)=b_{0}^{\tau }\left(u\right)+\sum_{k=1}^{p}b_{k}^{\tau }\left(u\right)\theta_{k},$$

$$\log\left(k\left(u\right)\right)=b_{0}^{k}\left(u\right)+\sum_{k=1}^{p}b_{k}^{k}\left(u\right)\theta_{k},$$

The precision matrix with parameter fields in the diagonal matrices is evaluated in a mesh as

$$T=diag\left(\tau \left(u_{i}\right)\right),\;\;K=diag\left(k\left(u_{i}\right)\right),$$

$$Q=T\left(K^{2}CK^{2}+K^{2}G_{1}+G_{1}^{T}K^{2}+G_{2}\right)T$$

The space–time SPDE model used in this study is represented as show below. This is by constructing Kronecker product model by first starting with the basis function represented as $x\left(u,t\right)=\sum_{k}\psi_{k}\left(u,t\right)x_{k}$ where each basis function is computed as a product of a spatial and a temporal basis function, $\psi_{i}\left(u,t\right)=\psi_{i}^{u}\left(u\right)\psi_{j}^{t}\left(t\right)$, thus the space–time SPDE (Ingebrigtsen et al., 2013). The temporal aspect of the model is based on an AR(2) process.

$$\frac{\partial }{\partial t}{\left(k{\left(u\right)}^{2}-\Delta \right)}^{\alpha /2}\left(\tau \left(u\right)x\left(u,t\right)\right)=w\left(u,t\right),\;\;\left(u,t\right)\in \Omega \times R$$

A model for seasonal variation where periodicity m=4 for a vector $\xi =(x_{1}\ldots ,x_{n}),n>m$ was obtained assuming the sum $x_{i}+x_{i+1}+\cdots +x_{i+m-1}$ are dependent Gaussian with precision *τ*. The density for *x* was derived from the n−m+1 increments as

$$\pi \left(x|\tau \right)\propto \tau \frac{\left(n-m+1\right)}{2}\exp\left\{-\frac{\tau }{2}\sum {\left(x_{i}+x_{i+m-1}\right)}^{2}\right\}$$

$$=\tau \frac{\left(n-m+1\right)}{2}\exp\left\{-\frac{1}{2}x^{T}Qx\right\}$$

The precision parameter *τ* is represented as θ=logτ and the prior defined on *θ*.

The posterior mean for each specified year-season of prediction was mapped as continuous maps at 1 × 1 km^2^ spatial resolution and further classified using the WHO guide for assessing severity of wasting by prevalence ranges among children less than 5 years of age (de Onis and Blossner, 2003). Wasting prevalence ranges among children under the age of five years identified by WHO are; (1) less than 5% for low risk (acceptable range); (2) 5% to less than 10% for medium risk (alert stage), 10% to less than 15% for high risk (serious stage), and 15–20% for very high risk (critical stage) and greater than 20% to represent very high (very critical) prevalence of wasting (de Onis and Blossner, 2003). These prevalence ranges are used to assess the severity of wasting as the basis for making public health decisions by establishing “trigger-levels” (de Onis and Blossner, 2003, Group, 1992). The population of wasted children was computed for each zone in Somalia by season in each year and prevalence class. This was obtained by multiplying the posterior mean proportion of wasted children at each 1 × 1 km^2^ pixel with the corresponding population of the under-fives at the same level provided by WorldPop population surfaces for season of the year to get the number of children wasted at 1 × 1 km^2^ pixel (Linard et al., 2010). Estimated numbers of children who were wasted, by season and WHO risk class were then summed up by zone. WorldPop models population distribution by assigning census population to pixels within an area based on weights derived from land cover classification. This is further refined by overlaying with detailed settlement extents to produce a gridded dataset of population distribution at 100 m resolution. Population distribution was then projected forward to 2010 using growth rates estimated by the UN Population Division (Linard et al., 2010).

To assess the predictive performance of the models, we generated a validation dataset by randomly selecting 10% of the data using a sampling algorithm which de-clusters over space and time. Four performance indices were then used to evaluate predictive performance and model fit: root-mean-square error (RMSE), mean prediction error, mean absolute prediction error and the correlation coefficient between the predicted and the observed values. Detailed methods on covariate selection, Bayesian hierarchical space–time modeling and validation procedures are given in Appendix 1.

## 3 Results

**Fig. 1:**
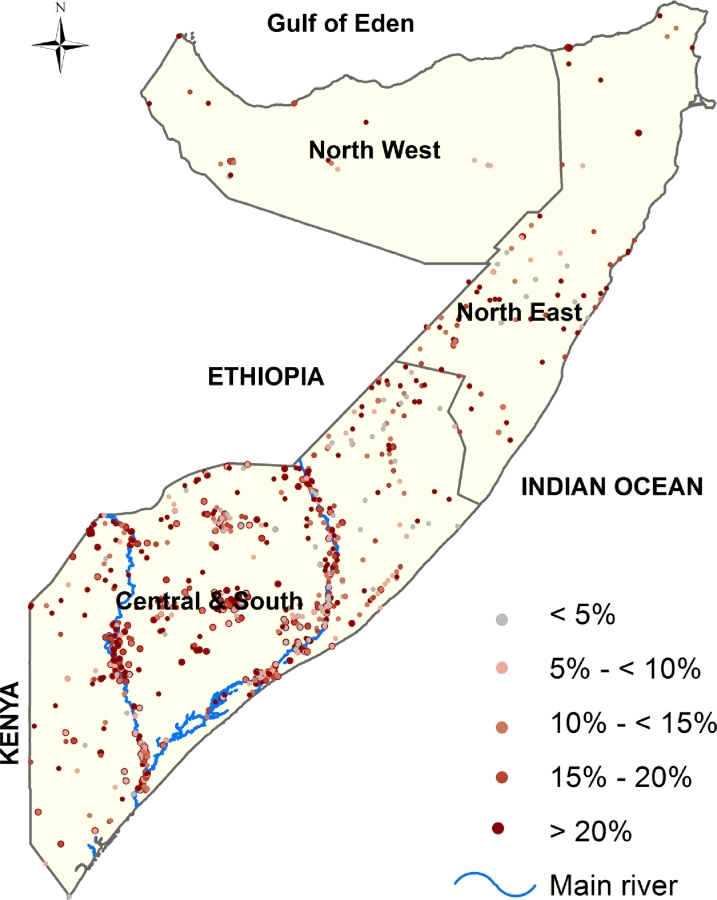
Map showing the distribution of clusters sampled for FSNAU nutrition surveys conducted between 2007 and 2010 in Somalia. The country is divided into three main zones: North West, North East and South Central. 78 clusters were sampled in North West zone, 85 clusters in the North East zone and 903 clusters in the South Central. The country's two main rivers, Juba and Shebelle are located in the South Central zone.

**Fig. 2:**
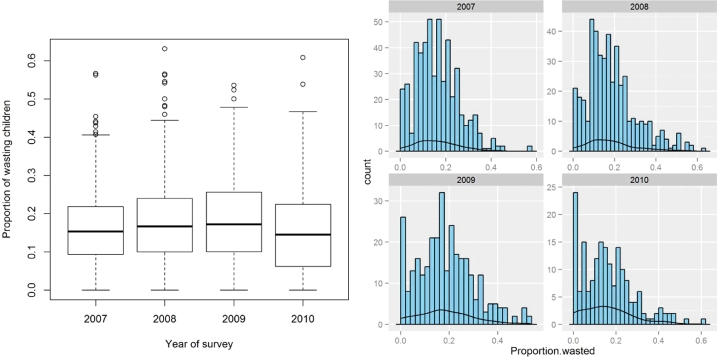
Box plots and histograms of the proportion of children aged 6 to 59 months wasted in Somalia from 2007-2010.

**Fig. 3:**
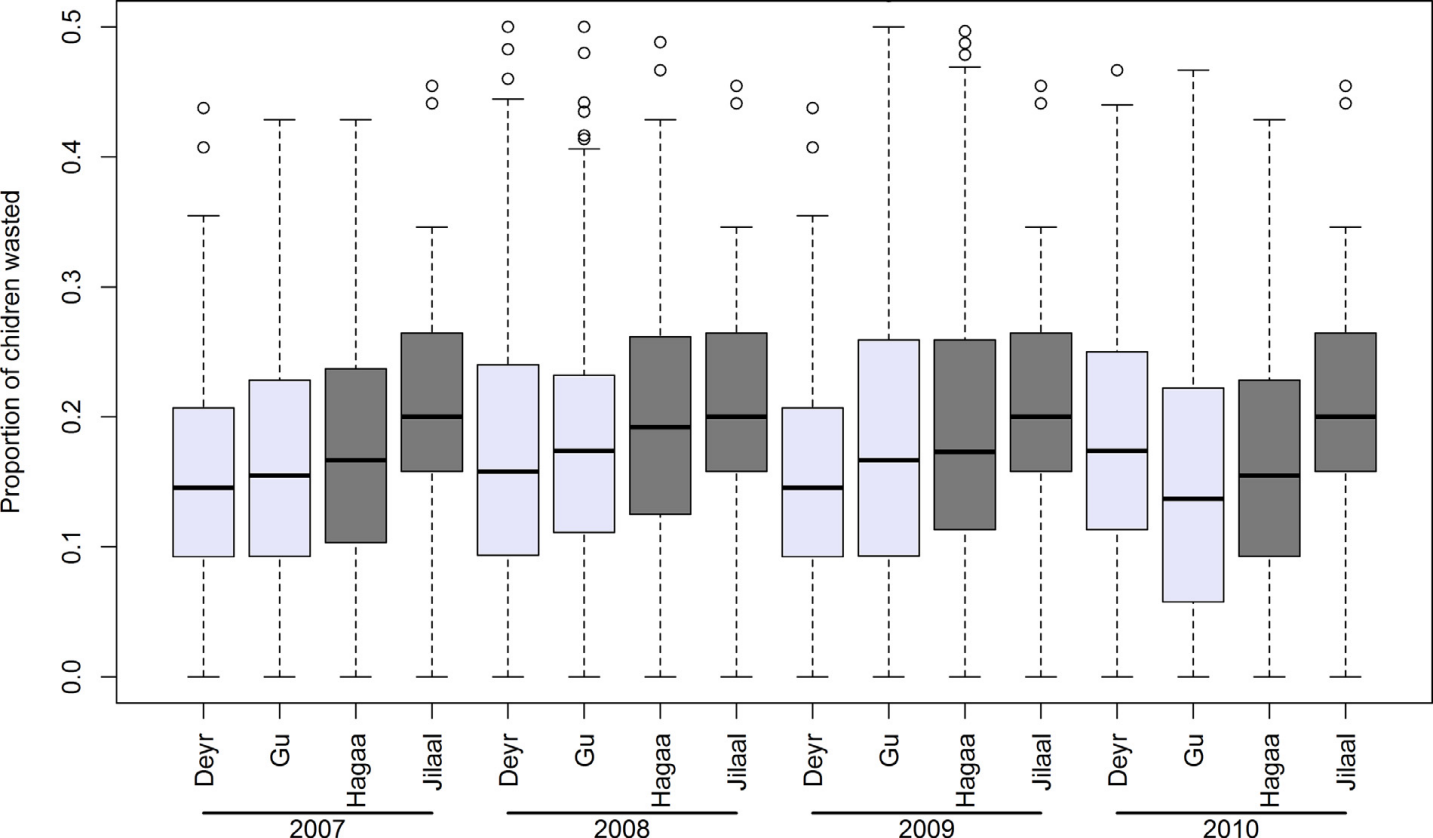
Seasonal variations of the proportion of children aged 6–59 months wasted in Somalia from 2007–2010. The April-June season is the main rainy season ‘Gu’; October–November is the short rainy season ‘Deyr’; July–September is the short dry season ‘Hagaa’; and the December–March is the long dry season ‘Jilaal’.

A total of 1066 unique survey locations sampled during the period of 2007–2010 were included in the analysis. Thirty six percent (36%) of the data was collected in 2007, 27% in 2008, 22% in 2009 and 14% in 2010 [Fig. 1]. Of 73,778 children, 15,735 (21%) were wasted (Table 1). The mean proportion of wasted children increased slightly from 2007 to 2009 and then decreased in 2010 in the observed dataset (Fig. 2). There was a clear seasonal variation: the prevalence of wasting was higher in the April–June season as compared to the October–November season from 2007 to 2009 but in 2010, the prevalence is higher in the October–November season than in the April–June season [Fig. 3]. The location of 1765 children from 34 clusters could not be accurately determined and were therefore excluded from the analysis.

Significant covariates used in the space–time geo-statistical model were; precipitation (OD = 0.999, credible interval (CrI) = (0.998–0.999), EVI (OD = 0.787, CrI = (0.727–0.851) and mean temperature (OD = 1.209, CrI = (1.163–1.258). Distance to water and urbanization were not significantly associated with wasting.

**Fig. 4:**
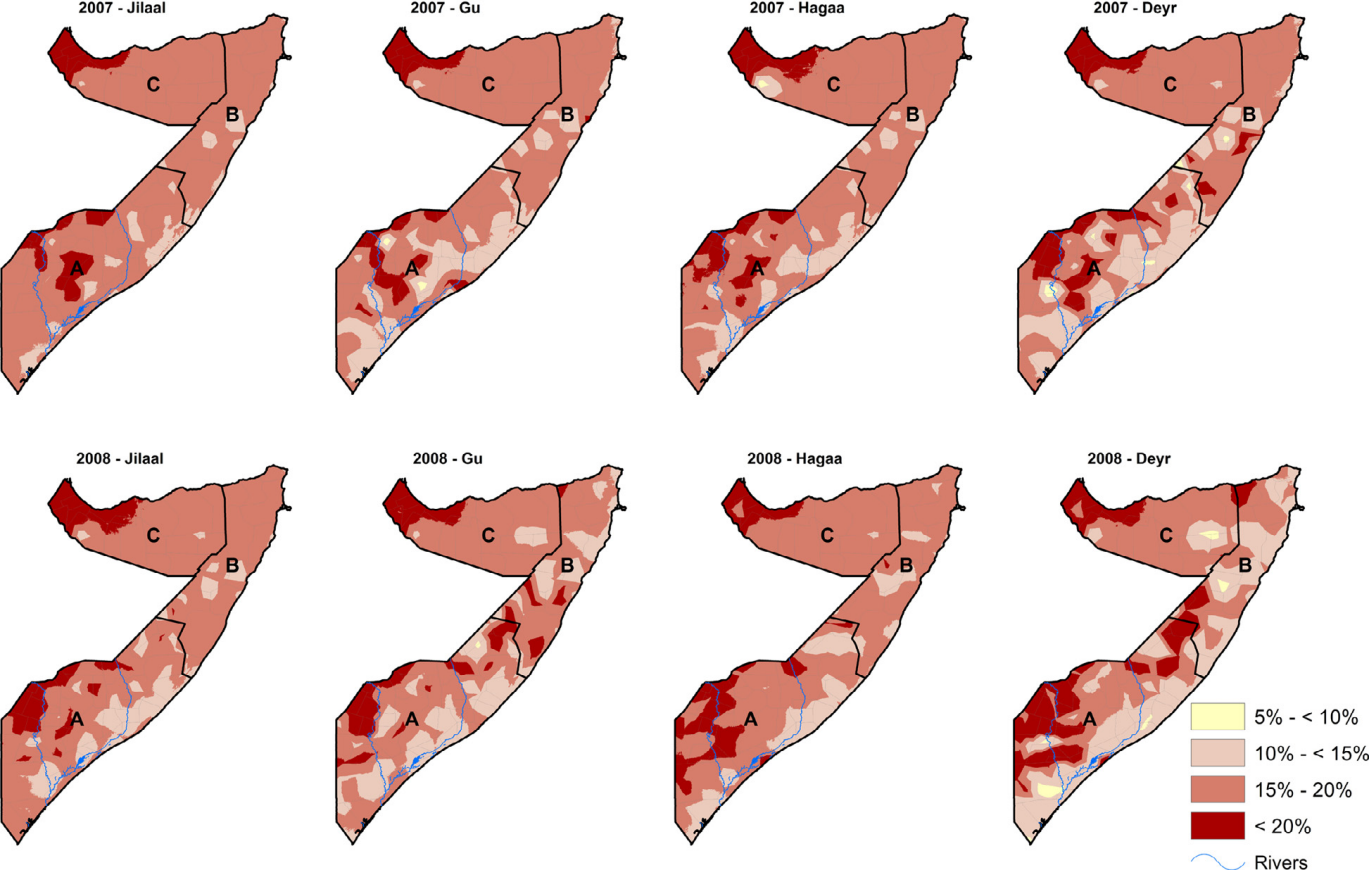
The predicted 1 × 1 km^2^ posterior prediction of wasting in Somalia for all the four seasons from 2007 and 2008 among children aged 6–59 months. Prevalence range of (5% to < 10%) represents ‘alert’ status, (10% to <15%) is ‘serious’, 15–20% is ‘critical’ and >20% is ‘very critical’ according to the WHO classification. The lowest prevalence of wasting in Somalia was above the acceptable level (5%) prevalence. A = South Central zone, B = North East zone, C = North West zone.

**Fig. 5:**
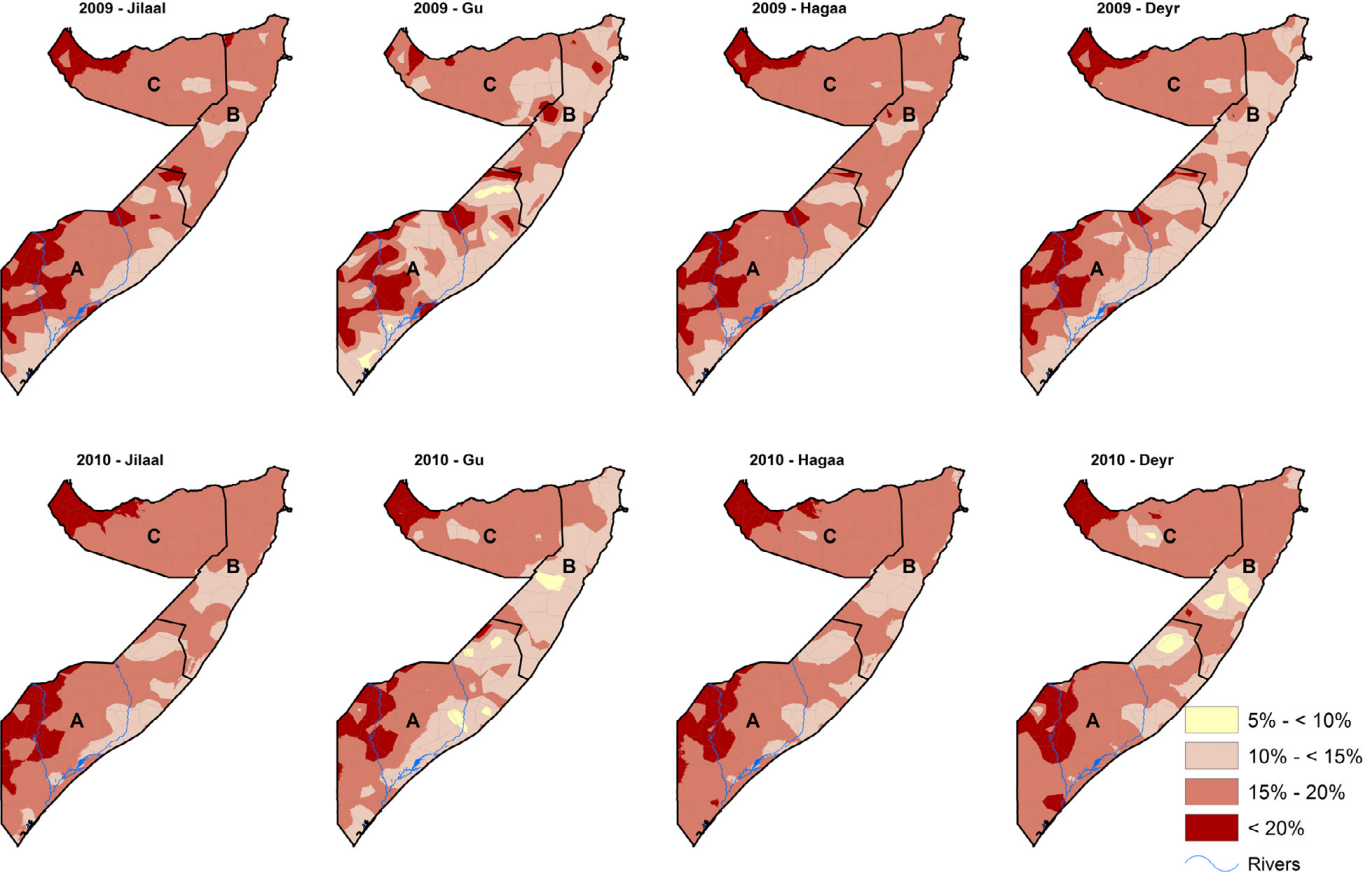
The predicted 1 × 1 km^2^ posterior prediction of wasting in Somalia for all the four seasons from 2009 to 2010 among children aged 6–59 months. Prevalence range of (5% to <10%) represents ‘alert’ status, (10% to <15%) is ‘serious’, 15–20% is ‘critical’ and >20% is ‘very critical’ according to the WHO classification. The lowest prevalence of wasting in Somalia was above the acceptable level (5%) prevalence. A = South Central zone, B = North East zone, C = North West zone.

The continuous posterior mean predictions of wasting in the four seasons of each year from 2007–2010 at 1 × 1 km^2^ spatial resolution were reclassified using the WHO classification for assessing severity of wasting among children less than five years of age [Figs. 4 and 5]. The prevalence of wasting was highest in the South Central zone followed by North West and lowest in North East. Overall, there was no change in wasting from 2007 to 2009 but a slight decrease in 2010.

**Fig. 6:**
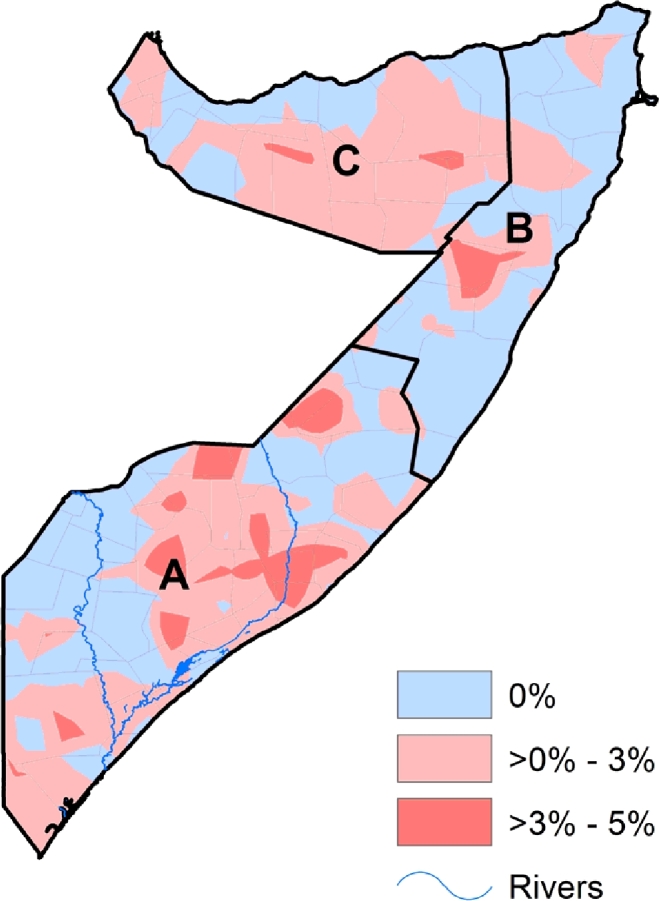
The mean difference of the prevalence of wasting during the dry and wet seasons among children under the age of five years in Somalia. The change in prevalence was computed by subtracting the prevalence of wasting during the wet season from the prevalence during dry season. A = South Central zone, B = North East zone, C = North West zone. The country's two main rivers, Juba and Shebelle are located in the South-central zone.

**Fig. 7:**
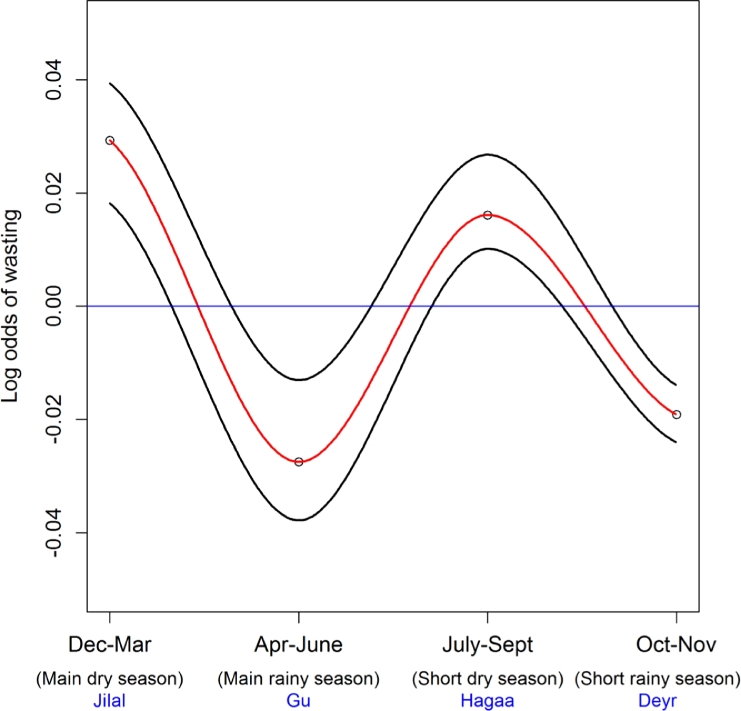
Seasonal patterns of wasting in children aged 6–59 months in Somalia. The figure shows the effect size of the four main seasons in Somalia on wasting: December to March is the ‘Jilaal’ season, a harsh dry season; ‘Gu’ which is the main rainy season from April to June; from July to September is the second dry season, the ‘Hagaa’; and the short rainy season known as ‘Deyr’ from October to December. The red line represents the mean and the black line shows the credible intervals. (For interpretation of the references to color in this figure legend, the reader is referred to the web version of this article.)

The mean difference of the prevalence of wasting between the dry and wet seasons ranged from 0% to 5%. The change in the prevalence varied from one region to the other while there were some regions with minimal change [Fig. 6]. Fig. 7 shows the effect size of seasonal variation on the prevalence of wasting. Of the two dry seasons, the odds of wasting were higher during the main dry season, which is in December–March (LOD = 0.04), as compared to the short dry season in July–September (LOD = 0.03). In the two rainy seasons, the odds of wasting was higher during the short rainy season during the months of October–November (LOD = −0.02) as compared to the main rainy season in April–June (LOD = −0.03).

The results show that there was no region in Somalia with acceptable levels of wasting of <5% prevalence as defined by the WHO. Table 2 shows the estimated numbers of children under five years of age that were wasted by year-season and prevalence class according to WHO classifications. Although the numbers of wasted children were highest in 2008 and 2009, estimated cases declined in every class between 2007 and 2010. By 2010 during the long dry season when wasting rates were highest, approximately 290,000 children were estimated to be wasted in Somalia, of which about 211,000 (73%) lived in areas that, according to the WHO, would be classified as having critical or very critical levels of wasting. The majority of these children were in the South Central zone where about 179,000 (62%) of wasted children and 104,000 (49%) of those from areas classified as having critical or very critical levels of wasting lived (Table 2).

Based on the predictions to the 10% hold-out dataset, the root mean square error (RMSE) was 0.136; mean error 0.002; absolute mean error 0.105 and the correlation coefficient 0.668. Maps of the posterior standard deviation from the mean can be found in Appendix 1 [Fig. SI 1].

## 4 Discussion

In this study, we used geostatistical models that combine survey data with climatic and seasonal determinants to estimate the prevalence of wasting and effect of seasonality using data on children under the age of five years assembled from 2007–2010 in Somalia. We found that the rate of wasting was generally at ‘critical’ (15%–20%) levels throughout the country, with several areas within the ‘very critical’ (>20%) range. Using a novel approach, we implemented two models to analyze the prevalence of wasting among children under the age of five years in two dry and two wet seasons and determine the separate effect of seasonality on the predicted estimates. To achieve this, we first let the temporal effects be informed by both the year and season of the survey to determine the prevalence of wasting in the four seasons in each year of survey. We then used a second approach whereby the temporal effect was dictated by the year of survey only and the four seasons within each year were used to fit seasonality in the model framework.

We observed minimal variation of wasting across years, but a clear seasonal variation with a relative rise during the dry seasons and reduction during the rainy seasons was revealed. The rate of wasting was highest during the main dry season in the December–March period when compared to the short dry season. During rainy seasons, the risk was higher in the main rainy season in April–June when compared to the short rainy season in October–November. The regions that showed consistent high levels of wasting in the four years were Gedo and Bay in South Central zone. Over time, the largest reduction was observed in the central regions compared to other parts of the country.

In Somalia, drought is estimated to account for 30% of the burden of natural disasters but over 80% of the population is affected (Shukla, 2014). Much of the country is dependent on rain-fed agriculture, which makes it particularly susceptible to climate variability. Almost 70% of the population is engaged in agricultural work, accounting for 65% of the gross domestic product, with livestock representing 40% of the GDP and 65% of the export earnings. The impacts of drought on agriculture and water resources have direct negative effects on the nutritional status in children (Below et al., 2007). These impacts are exacerbated by the generally high vulnerability of the local population and enhanced by prevailing local and external economic and political conditions (Olang et al., 2013), which may be accompanied by disease (Caulfield et al., 2004).

Wasting in Somalia is linked to high seasonal and inter-annual variability in rainfall. Studies in Gambia report seasonal fluctuation of wasting between 4% and 10% while in Niger the fluctuation was from 7% to 17% in chidren (Chotard et al., 2010, Loutan and Lamotte, 1984). Food insecurity which is the main drive of malnutrition has been shown to be linked to inter-annual variability in rainfall in most of the part of sub-Saharan Africa (Loutan and Lamotte, 1984). In Somalia, the mean monthly rainfall per year from 2007–2010 ranged from 2 to 104 mm. In general, a seasonal rainfall higher than 500 mm in sub-Saharan Africa is required to sustain healthy agriculture, highlighting the tenuous nature of agro-pastoral livelihoods in many parts of Somalia (Olang et al., 2013).

Livelihoods are threatened by increasingly erratic climatic conditions, competition of resources and security instability (FAO, 2010). For example, Gedo, the region that was observed to have a high prevalence of wasting, has three main rural livelihood zones; pastoral, agro-pastoral and riverine. This region has been adversely affected over time by the cumulative effects of extended conflict and recurrent poor rains (FAO, 2010) resulting in livelihood disruption, including loss of livestock and crop failure and culminated in a persistent emergency situation for a majority of the population (FAO, 2010, FAO, 2011).

Central Somalia, comprising the two regions of Galgadud and South Mudug, showed a remarkable reduction in wasting. This region is made up of four main livelihood zones; pastoral Addun and Hawd; the pastoral and fishing Coastal Deeh and the agro-pastoral Cowpea Belt. The Hawd and Addun pastoral communities extend across Galgadud, Mudug and the southern Nugaal regions while the Coastal Deeh extends from the coast of Shebelle through Galgadud up to Allula, cutting across the Central and Northeast regions. The Hawd and Addun pastoral livelihood communities were reported to have experienced a good April–June rainfall performance in 2010 (FAO, 2011). The coastal Deeh livelihood zone was also noted to have had an increase in small ruminant herd sizes in the same season (FAO, 2010). As a result, there was a significant pasture regeneration and farmers took advantage of the rising levels of the rivers (the Juba and Shebelle) to irrigate crops. This made cereal and livestock production possible, which may have cushioned the communities in this region from the effects of preceding drought and hunger (FAO, 2010).

The prevalence of wasting determined through nutrition surveys needs to be interpreted in relation to emergency levels and seasonal changes (Mason et al., 2010). This study has indicated that wasting varies widely according to spatial location, season and year-to-year conditions. Understanding the typical seasonal fluctuation is useful in assessing the severity of wasting at a particular location and time and this information can be used to predict the rates early enough in dry seasons for timely intervention (Chotard et al., 2010). For example the peaks of wasting in this study occurred during the dry seasons with the highest prevalence during the long dry season in December–March, which might have had an elevated effect on the prevalence in the long rainy season from April to June (Chotard et al., 2010). This type of information can be used during emergency humanitarian interventions, which involve distribution of food aid, setting up community mobilization, stabilization centers (SCs), targeted supplementary feeding programmes (TSFP), outpatient therapeutic feeding programmes (OTPs) for the management of severe acute malnutrition in Somalia (Matunga and Bush, 2014). Furthermore, the observed strong inter-seasonal variations, which in the study are stronger than the inter-annual patterns, highlight the limitation of infrequent national household surveys in estimating the burden of wasting, and the need for more frequent data which reflect the short term seasonal effects.

There are limitations to this study. The household survey data used in this study was aggregated by cluster due to the availability of coordinates at this level only and therefore did not account for child and household heterogeneity. In addition, rainfall is acutely seasonal in Somalia resulting in the likelihood of shifts in the start and end of the dry or wet seasons between years, depending on the climatic anomalies at the time. The assumption of fixed seasonal months may therefore lead to mis-classification of the wet and dry seasons, but due to the short temporal range of our study, is likely to have a minimal impact on our results.

## 5 Conclusion

Modeling of survey data together with climatic and other determinants reveals that wasting was at critical and very critical levels almost throughout the country prior to 2011**.** There were minimal annual fluctuations from 2007 to 2010 in Somalia, but a marked seasonality. Understanding seasonal fluctuation of wasting can be used to implement focused and timely interventions at a specific location and time to avert its devastating effects on children. Nutritional surveys should therefore be designed and implemented to allow for the seasonal disaggregation of data.

## Contributors

DKK, JAB, AMN and N-BK were responsible for the concept and design of the study. DKK led the development of the model, data assembly, analysis and interpretation of results. AMN, JAB and N-BK checked the statistical analysis, contributed to the methodology and assisted with the interpretation of the results. GMM and EOO were responsible for conducting the surveys, cleaning and archiving the data. All authors contributed to critical revisions for important intellectual content and contributed to the final submission. The paper is published with the permission of the Director, KEMRI.

## Competing interests

None declared.

## Figures and Tables

**Table 1:** Description of the household survey data used in this study (FSNAU 2007–2010).

Zone	Region	Number of clusters	Number of children examined	Number of children wasted	Percent wasted
**North East**	Bari	9	756	174	23.02
Mudug	61	6188	1055	17.05
Nugaal	24	1673	322	19.25
**North West**	Awdal	26	862	177	20.53
Sanaag	14	412	97	23.54
Sool	3	142	24	16.90
Togdheer	12	673	124	18.42
Woqooyi Galbeed	23	2465	480	19.47
**South Central**	Bakool	75	3534	1330	37.63
Banadir	1	51	11	21.57
Bay	98	5568	1798	32.29
Galgaduud	77	5831	879	15.07
Gedo	111	6985	2616	37.45
Hiraan	142	10,743	2085	19.41
Juba Dhexe	77	5253	960	18.28
Juba Hoose	71	5560	926	16.65
Shebelle Dhexe	101	7650	1322	17.28
Shebelle Hoose	141	9432	1355	14.37
**Total**	**18**	**1066**	**73,778**	**15,735**	**21.33**

**Table 2:** Estimates of the number of children wasted during the four seasons from 2007 to 2010 in Somalia. This was obtained by multiplying the posterior mean proportion of wasted children at each 1 × 1 km pixel with the corresponding population of the under-fives provided by WorldPop population grids (Linard et al., 2010). The results were then summed up by season and WHO prevalence class for each zone. The April–June season is the main rainy season ‘Gu’; October–November is the short rainy season ‘Deyr’; July–September is the short dry season ‘Hagaa’; and the December–March is the long dry season ‘Jilaal’.

			Zone name		
WHO prevalence class	Year of survey	Season	South Central	North East	North West
Alert (5%–<10%)	2007	Jilaal	<50	<50	<50
	Gu	<50	<50	<50
	Hagaa	<50	<50	713
	Deyr	696	<50	<50
2008	Jilaal	<50	<50	<50
	Gu	<50	<50	<50
	Hagaa	<50	<50	<50
	Deyr	774	<50	<50
2009	Jilaal	<50	<50	<50
	Gu	2,902	<50	<50
	Hagaa	<50	<50	<50
	Deyr	<50	<50	<50
2010	Jilaal	<50	<50	<50
	Gu	5449	142	<50
	Hagaa	<50	<50	<50
	Deyr	1,580	493	<50
Serious (10%–<15%)	2007	Jilaal	10,432	2,469	723
	Gu	27,995	2,841	1,082
	Hagaa	36,033	2,763	11,157
	Deyr	62,488	4,178	3,451
2008	Jilaal	66,112	2,262	3,841
	Gu	70,170	3,373	710
	Hagaa	80,547	1,445	630
	Deyr	75,239	2,771	6,438
2009	Jilaal	80,439	1,559	378
	Gu	72,928	3,694	1,717
	Hagaa	76,196	1,447	50
	Deyr	76,033	1,964	26
2010	Jilaal	74,787	3,770	20
	Gu	68,289	7,742	5,362
	Hagaa	41,826	5,813	3,797
	Deyr	23,748	6,501	6,319
Critical (15%–20%)	2007	Jilaal	146,467	27,892	53,562
	Gu	104,918	27,698	52,798
	Hagaa	121,632	27,933	37,643
	Deyr	75,470	26,244	49,172
2008	Jilaal	95,155	29,732	50,026
	Gu	81,592	20,587	53,818
	Hagaa	68,172	24,529	54,352
	Deyr	53,447	20,206	47,867
2009	Jilaal	65,327	25,123	57,530
	Gu	53,700	25,139	56,725
	Hagaa	73,117	31,014	59,582
	Deyr	70,780	30,038	59,321
2010	Jilaal	84,728	28,047	61,499
	Gu	76,227	23,035	53,061
	Hagaa	121,669	25,291	57,062
	Deyr	138,962	23,237	51,992
Very critical (>20%)	2007	Jilaal	30,349	<50	18,714
	Gu	57,915	71	18,887
	Hagaa	19,200	<50	17,886
	Deyr	28,497	918	19,766
2008	Jilaal	12,746	<50	20,222
	Gu	20,097	9,740	20,912
	Hagaa	24,931	7,076	20,572
	Deyr	38,627	13,559	20,142
2009	Jilaal	30,683	7,056	19,571
	Gu	51,120	6,026	17,517
	Hagaa	28,053	<50	18,178
	Deyr	28,288	<50	17,479
2010	Jilaal	19,540	<50	17,566
	Gu	22,962	<50	17,247
	Hagaa	19,783	<50	17,739
	Deyr	32,080	<50	17,888

## Appendix Supplementary materials

Application 1 Image, application 1
