# Supplementary material for: Space–time mapping of wasting among children under the age of five years in Somalia from 2007 to 2010
Source: Spat Spatiotemporal Epidemiol. 2016 Feb;16:77–87. doi: 10.1016/j.sste.2015.12.002 (PMC4813094; doi:10.1016/j.sste.2015.12.002)
Supplement: Application 1 [file mmc1.docx]

**Space-time mapping of wasting among children under the age of five years in Somalia from 2007 to 2010**

**SI 1: Covariate processing and selection**

A set of five geographical covariates were examined; Precipitation, Enhanced Vegetation Index (EVI), Temperature, Distance to water bodies and Urbanization. Precipitation and temperature rasters were derived from the monthly average rasters obtained from WorldClim website and were summarized to get the mean rainfall and mean temperature surfaces by season^1^. The EVI surface was derived from the MODerate-resolution Imaging Spectroradiometer (MODIS) sensor imagery ^2^ while the urbanization surface was obtained from Global Rural Urban Mapping Project (GRUMP)^3^.

We began the process of covariate selection with extracting the values of the selected covariates from the geographical surfaces to survey locations in the dataset using ArcGIS 10 Spatial Analyst (ESRI Inc. NY, USA) tool. Then best generalized linear approach was used to generate minimum adequate set of covariates that have a significant effect on wasting to be used in Geo-statistical model. The function “bestglm” was used as implemented in R-Project version 3.0.1 package. This function selects the best subset of the input covariates for the GLM family^4^. We used the Bayes Information Criterion (BIC) to select the significant covariates for the study because it has been shown that BIC often selects more parsimonious models than the AIC^4^. A uniform prior of the model of fixed size implemented in $BIC_{\gamma}$ was used (Equation 1).

$BIC_{\gamma}=D+k\log(\mathrm{n})+2\gamma\log\left(\begin{matrix} p \\ k \end{matrix}\right)$ (1)

This is where $\gamma$ is an adjustable parameter, p is the number of possible input covariates not counting the bias or intercept term and $k$ is the number of the parameters in the model^4^.

**SI 2: Space-time Bayesian Geo-statistical model**

We implemented a Bayesian hierarchical space-time model through SPDE approach using R-INLA library to produce continuous maps of the risk of wasting at 1 x 1 km spatial resolution and predicting to each season of the year of study from 2007 to 2010^5^. In our case the observed prevalence of wasting in a particular cluster and time is assumed to have a distribution that belongs to the exponential family and the parameters of the family ( ) are linked to a structured additive predictor $\eta$ through a link function such that the linear predictor $\eta$ is defined. Therefore, our hierarchical space-time binomial model of the prevalence of wasting was represented as the realization of a spatial-temporal process of wasting among children under the age of five years at the survey location, survey date, significant covariates at sampled locations, and the measurement error defined by the Gaussian white noise process. This can simply be denoted as,

(2)

This equation defines a hierarchical model where $\beta_{0}$ is the intercept, $\beta$ denotes the linear regression coefficients for the covariates$(Cov)$. The function $f_{k}$ is the sum of smooth functions defining the random effect of the clusters where the regression coefficients vary with $K$ values and $w_{k}$ is a vector of sampled clusters. is a semi-parametric function defining the spatio-temporal random effect in the model. These components form the non-observable latent field defined as $\theta=\left\{\beta_{0},\beta,f\right\}$ where $\beta$ and $f$ are the covariates and smooth functions included in the linear predictor with their appropriate priors $(\psi)$ .The latent field is characterized by a joint normal Gaussian multivariate distribution with mean $0$ and precision matrix $Q(\psi)$ , i.e., $\theta\sim N(0,\mathrm{Q}^{-1}(\psi))$ . Each observation $y_{i}$ depends on a linear combination of a subset of the elements of $\theta$ defined as:

$y_{i}|\theta,\psi\sim p\left(y_{i}|\sum_{j} A_{ij}\theta_{j},\psi\right)$ (3)

This is where $A_{ij}$ is the generic element of the observed matrix $A$ defined by the SPDE approach. This SPDE is formulated as a link between Gaussian random fields (GRFs) and the Gaussian Markov Random Fields (GMRFs)^6^. The spatio-temporal covariance function and the dense covariance matrix of the Gaussian field are replaced by a neighborhood structure and a sparse precision matrix respectively that together define a GMRF^7^. This finite-dimensional GMRF that substitutes infinite-dimensional GRF can be expressed as shown in Equation 4.

$x(u)=\sum_{i=1}^{n} \psi_{i}(u)w_{i}$ (4)

This SPDE-formulation is motivated by computational benefits and also introduces a new class of spatial models^8^. In this SPDE approach, a non-stationary model was used and achieved by modifying the SPDE to obtain the GRFs with defined dependence structure and is expressed as

$(k(\mathrm{u})^{2}-\Delta)(\tau(\mathrm{u})\mathrm{x}(\mathrm{u}))=\mathrm{w}(\mathrm{u}),$ $u\in\Omega,$ (5)

In the current version of the SPDE package as implemented in^6^, a non-stationary model defined via spatial varying $k(\mathrm{u})$ and $\tau(\mathrm{u})$ is available for the case$\alpha=2$ . The $\mathrm{logk}(\mathrm{u})$ and $\log\tau(\mathrm{u})$ are defined as linear combinations of basis functions,

$\log(\tau(\mathrm{u}))=\mathrm{b}_{0}^{\tau}(\mathrm{u})+\sum_{k=1}^{p} b_{k}^{\tau}(\mathrm{u})\theta_{k},$ (6)

$$\log(\mathrm{k}(\mathrm{u}))=\mathrm{b}_{0}^{k}(\mathrm{u})+\sum_{k=1}^{p} b_{k}^{k}(\mathrm{u})\theta_{k},$$

The precision matrix with parameter fields in the diagonal matrices is evaluated in a mesh as

$T=diag(\tau(\mathrm{u}_{i})),$ $K=diag(\mathrm{k}(\mathrm{u}_{i})),$ (7)

$$Q=T(\mathrm{K}^{2}\mathrm{CK}^{2}+\mathrm{K}^{2}\mathrm{G}_{1}+\mathrm{G}_{1}^{T}\mathrm{K}^{2}+\mathrm{G}_{2})\mathrm{T}$$

The space-time SPDE model used in this study is represented as show in equation 8. This is by constructing Kronecker product model by first starting with the basis function represented as $x(u,t)=\sum_{k} \psi_{k}(u,t)\mathrm{x}_{k}$ where each basis function is computed as a product of a spatial and a temporal basis function, $\psi_{i}(\mathrm{u},\mathrm{t})=\psi_{i}^{u}(\mathrm{u})\psi_{j}^{t}(\mathrm{t})$, thus the space-time SPDE^6^. The temporal aspect of the model is based on AR(2) process.

$\frac{\partial}{\partial t}(\mathrm{k}(\mathrm{u})^{2}-\Delta)^{\alpha/2}(\tau(\mathrm{u})\mathrm{x}(\mathrm{u},\mathrm{t}))=\mathrm{w}(\mathrm{u},\mathrm{t}),$ $(\mathrm{u},\mathrm{t})\in\Omega\times R$ (8)

A model for seasonal variation where periodicity $m=4$for a vector $\xi=(\mathrm{x}_{1}\ldots,\mathrm{x}_{n}),\mathrm{n}>\mathrm{m}$ was obtained assuming the sum $x_{i}+x_{i+1}+\ldots+x_{i+m-1}$ are dependent Gaussian with precision $\tau$ . The density for $x$ was derived from the $n-m+1$ increments as

$\pi(\mathrm{x}|\tau)\propto\tau\frac{\left(n-m+1\right)}{2}\exp\left\{-\frac{\tau}{2}\sum(\mathrm{x}_{i}+\mathrm{x}_{i+m-1})^{2}\right\}$ (9)

$=\tau\frac{\left(n-m+1\right)}{2}\exp\left\{-\frac{1}{2}x^{T}Qx\right\}$ (10)

The precision parameter $\tau$ is represented as $\theta=\log\tau$ and the prior defined on $\theta$ .


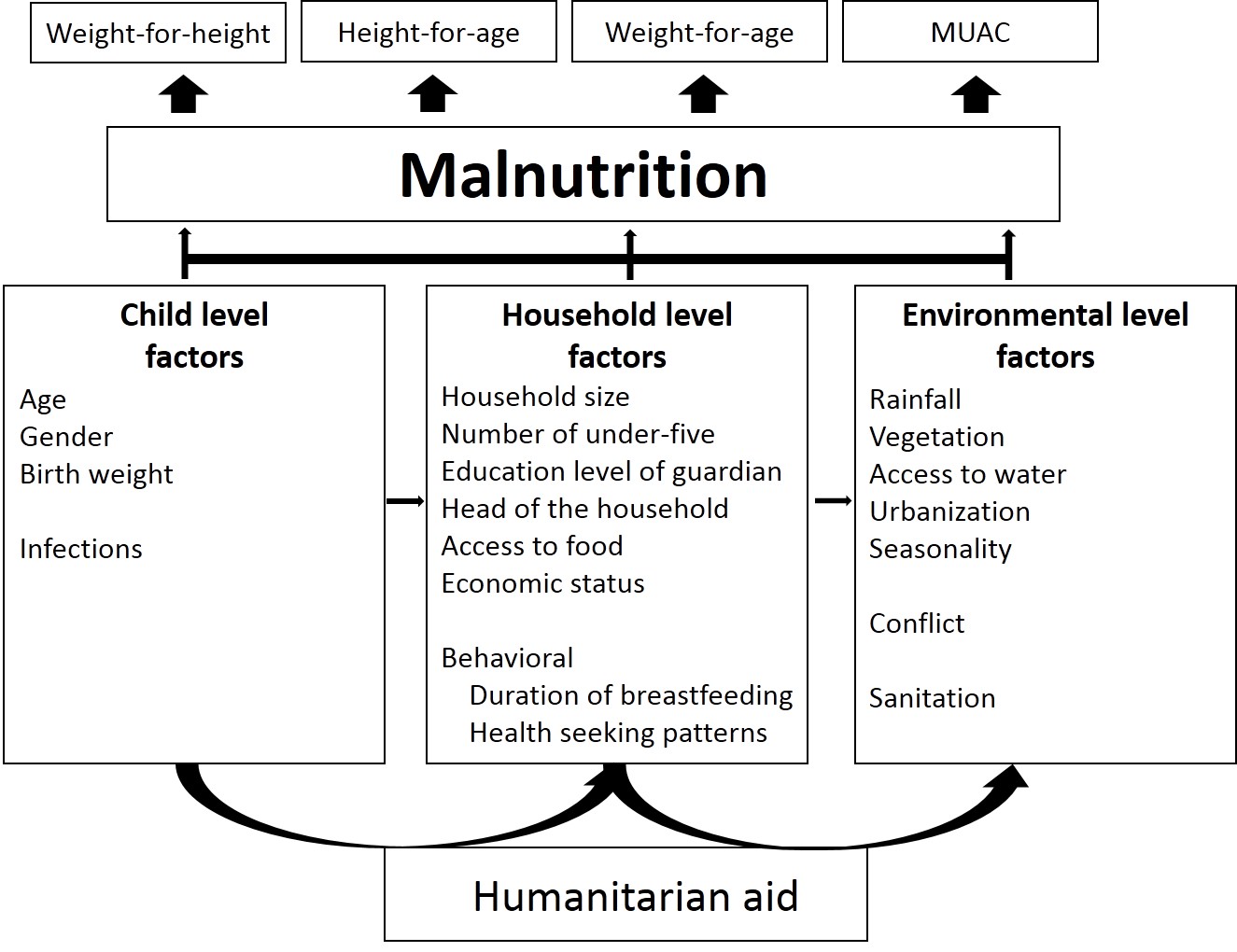


**Figure SI 1:** Conceptual framework of the potential predictors of malnutrition in Somalia.


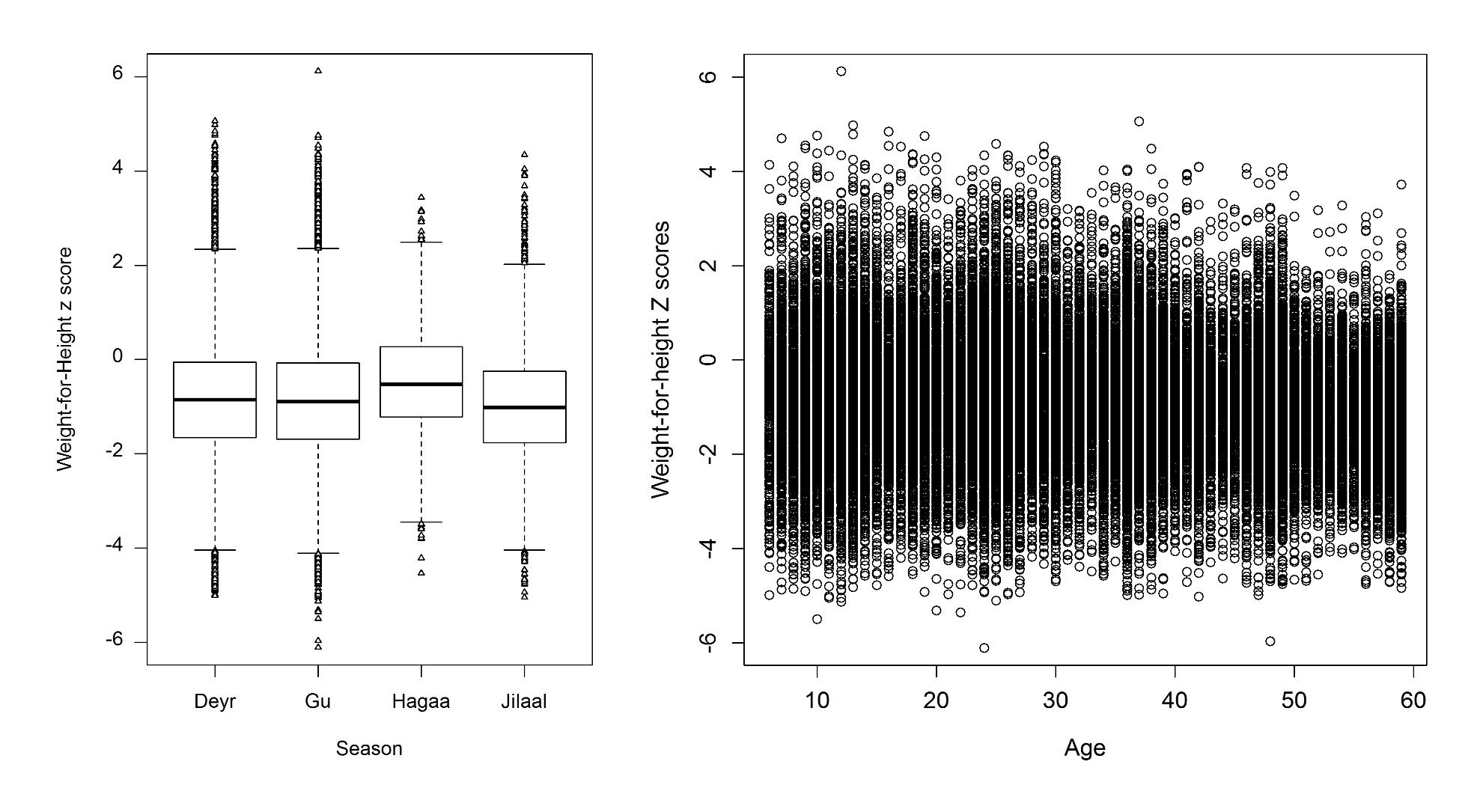


**Figure SI 2:** Seasonal variation of weight-for-height Z scores among children aged 6-59 months in Somalia


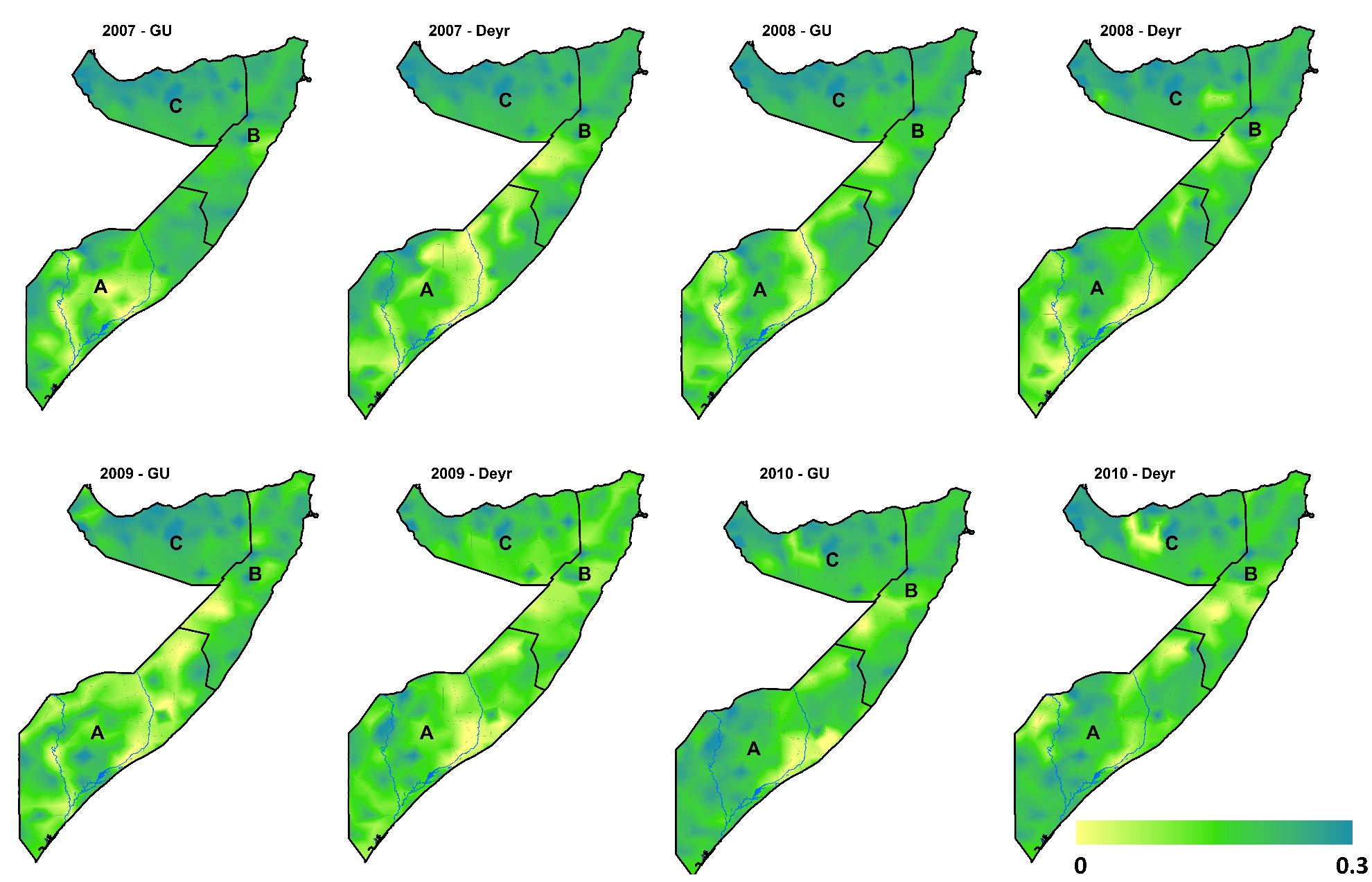
**Figure SI 3:** Continuous maps at 1 x 1 km spatial resolution of standard deviations from the predicted mean prevalence of wasting. A=South Central zone, B=North East zone, C=North West zone.


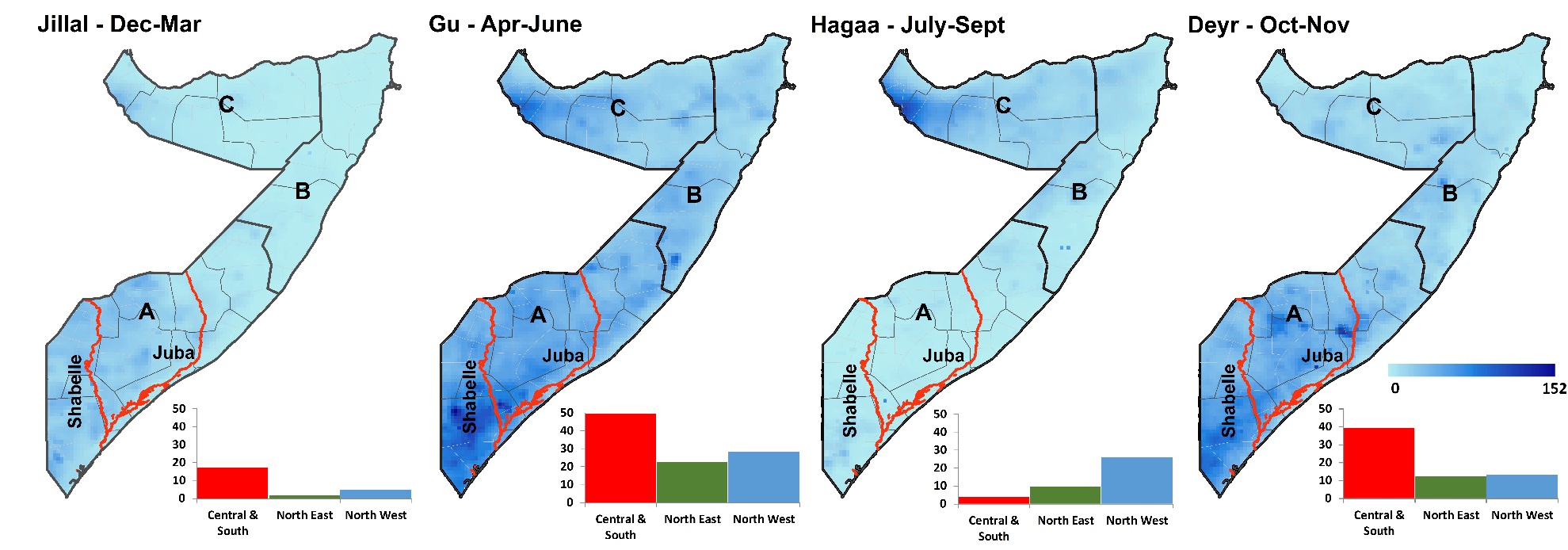


**Figure SI 4:** Spatial patterns of the synoptic mean rainfall (mm) from 2007 to 2010 for the four main seasons in Somalia: December - March; April - June; July – September; October to December. A=South Central zone, B=North East zone, C=North West zone. The red lines represent the two rivers in Somalia (Juba and Shebelle).


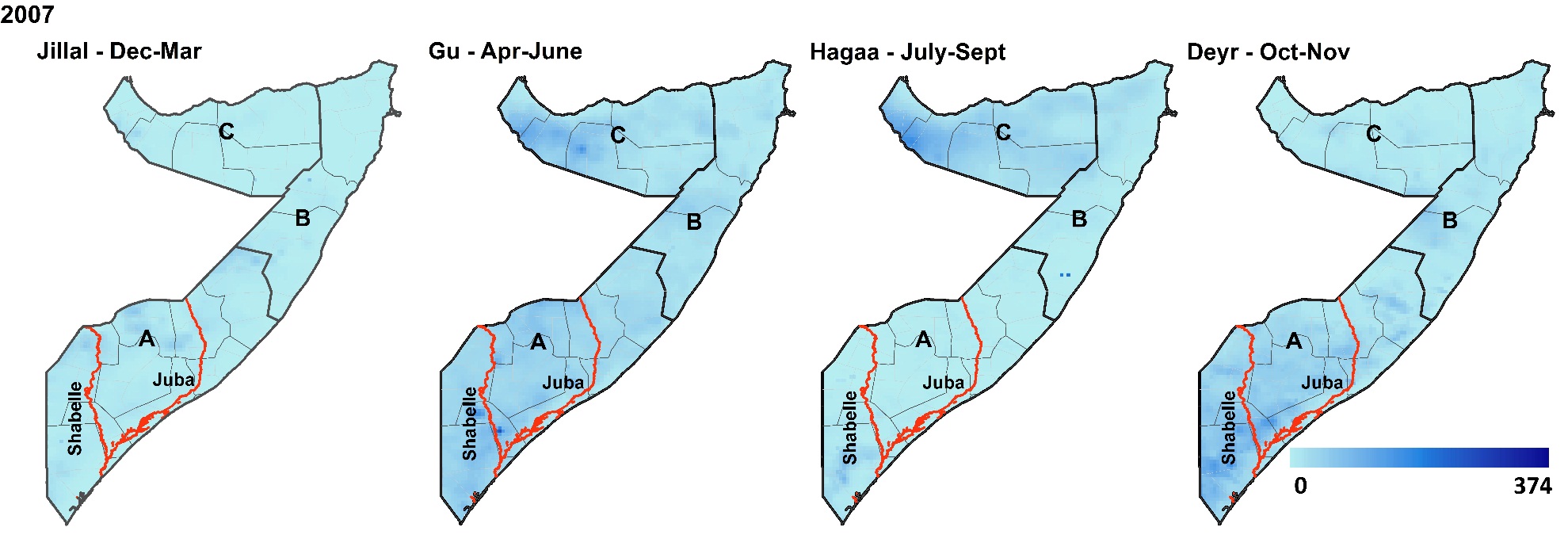


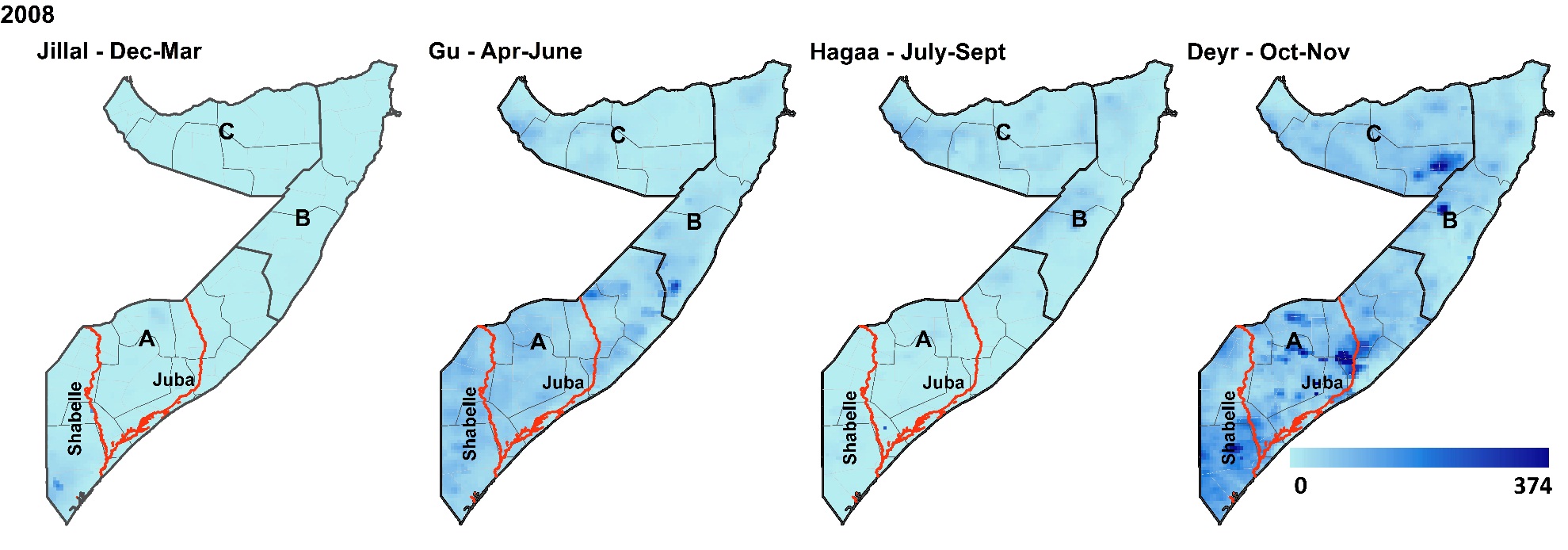


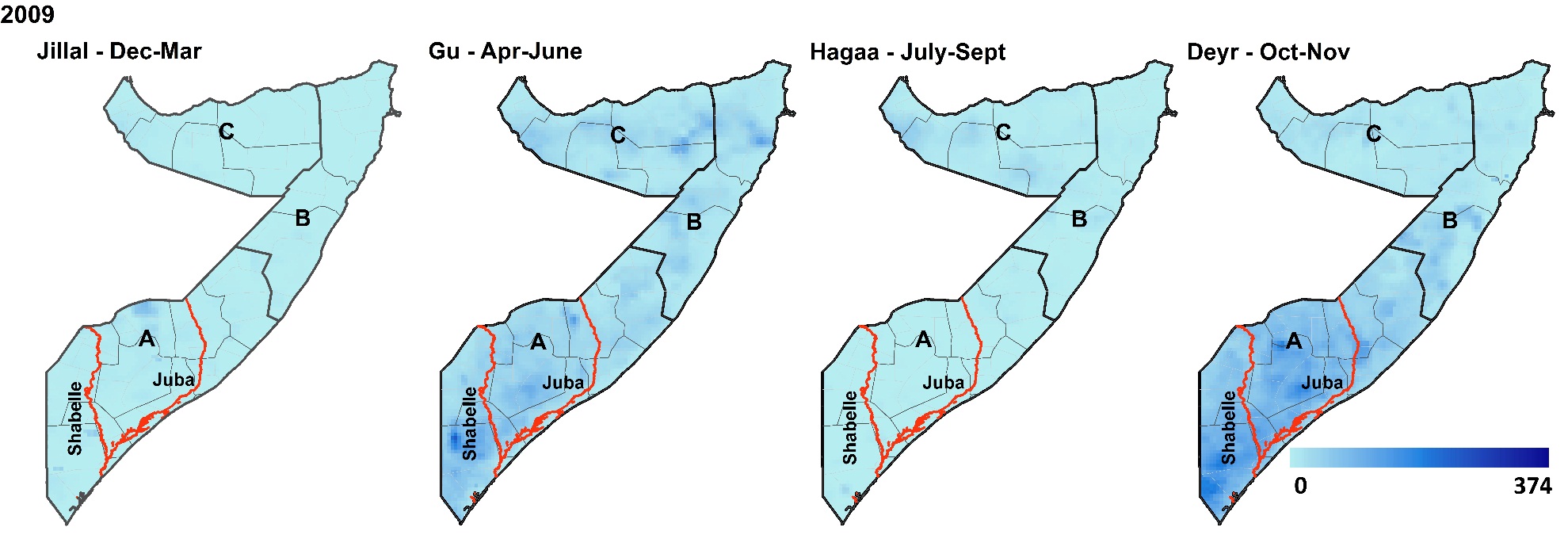


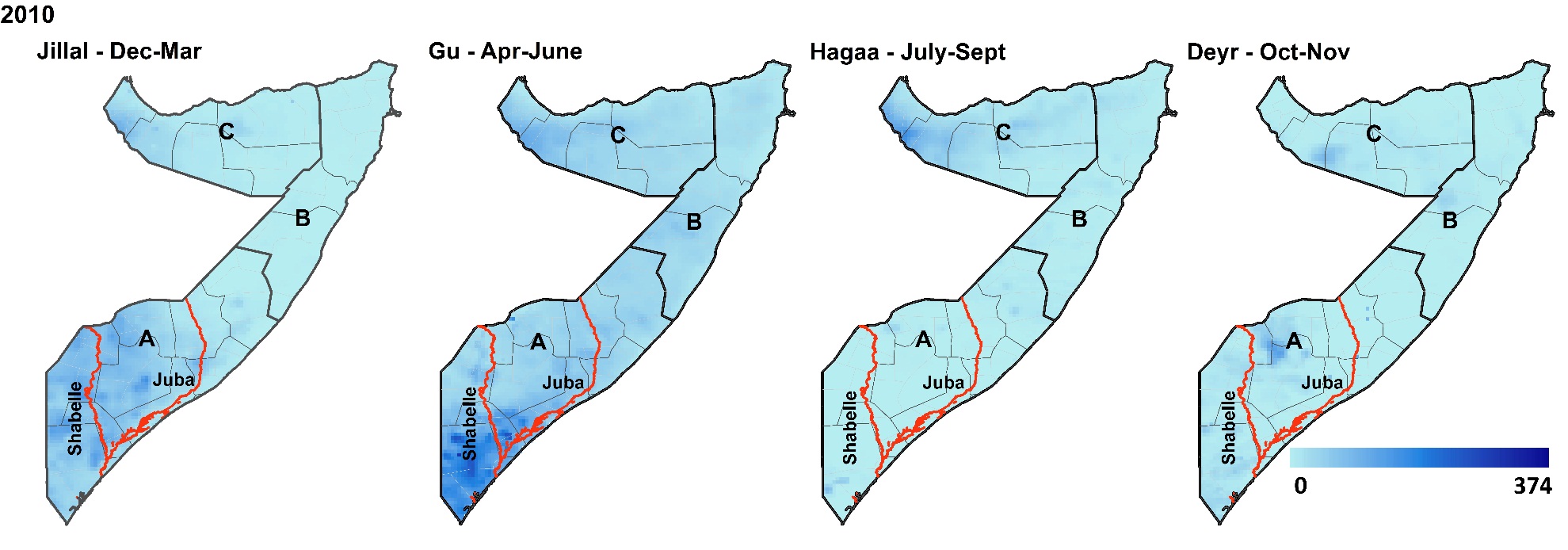


**Figure SI 5:** The seasonal mean rainfall per year from 2007 – 2010 in Somalia: December - March; April - June; July – September; October to December.

**References**

1. Murtaugh PA. Performance of several variable-selection methods applied to real ecological data. *Ecology Letters.* 2009;12(10):1061-1068.

2. Scharlemann JorP, Benz D, Hay SI, et al. Global data for ecology and epidemiology: a novel algorithm for temporal Fourier processing MODIS data. *PLoS One.* 2008;3(1):e1408.

3. Schneider A, Friedl M, Potere D. A new map of global urban extent from MODIS satellite data. *Environmental Research Letters.* 2009;4(4):044003.

4. McLeod A, Xu C. bestglm: Best Subset GLM. *URL* [*http://CRAN*](http://CRAN)*. R-project. org/package= bestglm.* 2010.

5. Rue Hav, Martino S, Chopin N. Approximate Bayesian inference for latent Gaussian models by using integrated nested Laplace approximations. *Journal of the royal statistical society: Series b (statistical methodology).* 2009;71(2):319-392.

6. Lindgren F, Rue H. Bayesian Spatial and Spatio-temporal Modelling with R-INLA.

7. Cameletti M, Lindgren F, Simpson D, Rue Hav. Spatio-temporal modeling of particulate matter concentration through the SPDE approach. *AStA Advances in Statistical Analysis.* 2013:1-23.

8. Ingebrigtsen R, Lindgren F, Steinsland I. Spatial models with explanatory variables in the dependence structure. *Spatial Statistics.* 2013.
